# Comparative Effectiveness of Electrical Stimulation and Aerobic Exercise in the Management of Erectile Dysfunction: A Randomized Clinical Trial

**DOI:** 10.4314/ejhs.v30i6.14

**Published:** 2020-11

**Authors:** Adamu Rislanu, Hassan Auwal, Danazumi Musa, Abdulahi Auwal

**Affiliations:** 1 Department of Physiotherapy, University of Maiduguri Teaching Hospital, Maiduguri, Borno State, Nigeria; 2 Department of Medical Rehabilitation (Physiotherapy), Faculty of Allied Health Sciences, College of Medical Sciences, University of Maiduguri, Maiduguri, Borno State, Nigeria; 3 Department of Physiotherapy, Federal Medical Centre Nguru, Yobe State, Nigeria; 4 Department of Physiotherapy, College of Health Sciences, Faculty of Allied Health Sciences, Bayero University, Kano, Nigeria

**Keywords:** Erectile dysfunction, Electrical stimulation, Aerobic exercise, International Index of Erectile Function

## Abstract

**Background:**

Electrical stimulation and aerobic exercise have been indicated to be beneficial in the management of erectile dysfunction individually. However, there is a scarcity of evidence comparing the two treatment approaches. This study investigated the effects of Electrical Stimulation (ES) compared with Eerobic Exercise (AE) in the management of individuals with Erectile Dysfunction (ED).

**Methods:**

This study was a single-blind parallel randomized clinical trial. Thirty (30) patients diagnosed with ED (Mean age of 39.17 ± 6.21 years) were recruited and randomized into two groups, A and B with 15 participants in each group. Group A received ES while Group B received AE. International Index of Erectile Function (IIEF-5) was used to assess the sexual functions of the participants at baseline and after 6 weeks of intervention. Within-group and between-group differences were analyzed using dependent and independent t-tests respectively.

**Results:**

The result indicated a significant difference between groups A and B [20.83 (1.83) Vs 14.33 (2.07), p=0.001] after 6 weeks of intervention. However, the mean effect was significantly higher in the ES group than in the AE group.

**Conclusion:**

The finding of this study indicated that ES is more effective than AE in the management of individuals with ED.

Trial Registration: Pan African Clinical Trial Registry (PACTR201906776769795)

## Introduction

The term male Erectile Dysfunction (ED) refers to a recurring and persistent condition where a man is unable to achieve or maintain an erection and complete sexual intercourse (1). ED is very common, and its prevalence as well as severity increases with age (2). A review of published studies found that estimates for the global prevalence of erectile dysfunction vary widely, ranging from 3% to 76.5%. In Nigeria, a recent study reported a high prevalence of ED (66.4%) among 378 male adults, with most of the participants having poor health-seeking behavior due to associated stigmatization (4).

Therapeutic options for men with ED are mainly administration of phosphodiesterase type 5 inhibitors, intracavernous injections of vasoactive agents (for example, prostaglandin El, papaverine/phentolamine, or triple-drug), intraurethral administration of prostaglandin El, and administration of centrally acting drugs (5,6). However, all of these methods treat the patient's problem temporarily, and patients are not cured of impotence. Patients typically remain dependent on these treatments for the remainder of their sexually active lives and for these reason, alternative effective treatment options are needed (6).

ED is usually of multifactorial origin ranging from physiologic, organic, psychogenic and endocrine factors (7). However, it has been recognized that the major cause of ED is atherosclerosis affecting the pelvic vasculature (1). It is known that atherosclerotic lesions prevent blood flow into cavernosal tissues resulting in ED (8). Although vascular factors predominate in the etiology of ED, conditions associated with reduced nerve and endothelium function, such as ageing, hypertension, smoking, hypercholesterolemia and diabetes, also alter the balance between contraction and relaxation factors (9). Almost any disease that may affect erectile function such as benign prostatic hyperplasia, prostate cancer, depression, and infectious diseases among others, alter the nervous, vascular and hormonal systems (10).

Considering the multiple causes of ED and the subsequent vascular or nervous affectation, it would be helpful to determine the most effective treatment between exercise and electrical stimulation of nerves. There has already been a report of the inverse relationship between physical activity levels and biomarkers of inflammation in both healthy individuals and subjects with cardiovascular conditions (11). Accordingly, recent studies have indicated the role of aerobic exercise in the management of erectile dysfunction (12–16). Aerobic exercise has been revealed to strengthen the cardiovascular system by making the heart stronger and the lungs more efficient (17). A stronger heart delivers more blood to the body with fewer beats, which also lowers blood pressure. Efficient lungs can transfer more oxygen into the bloodstream with each breath (17). Nevertheless, there is the need for a reliable alternative to aerobic exercise in case of contraindications such as electrocardiography changes, myocardial infarction, congestive heart failure, unstable angina, complete heart block, and uncontrolled hypertension. Moreover, exercise may also not be very effective if nervous affectation is implicated in the pathophysiology of individual's ED.

Recent studies have demonstrated the effectiveness of penile electrical stimulation in the management of ED (11,18–20). Also reported was a randomized control trial to find out the efficacy of magnetic stimulation of the cavernous nerve for the treatment of erectile dysfunction, which proved to be effective in producing increased intercorporal pressure and penile tumescence and rigidity (21). By applying electrical stimulation with appropriate stimulation parameters, the insufficiency of the carvenosal-smooth muscle can be treated (22). The muscle will be strengthened and the resulting increase of muscular mass and physical strength can lead to an improvement of the functioning of venous occlusion mechanism and enable the required filling of the corpus carvenosal bodies with the blood (22).

Considering the contraindications of electrical stimulation such as impaired sensation, insufficient blood flow and presence of a metallic implant, and also the aforementioned contraindications of exercise, in addition to multifactorial causes of ED, it will be important to determine the relative efficacy of aerobic exercise in comparison to electrical stimulation in the management of this condition. This will help in providing the patients with the most cost-effective and relatively safe choice among the two options according to their conditions and need. Furthermore, an extensive literature search indicated scarcity of studies that compared electrical stimulation with aerobic exercise in the management of individuals with ED. It was hypothesized that there will be no significant difference between electrical stimulation and aerobic exercise in the management of individuals with erectile dysfunction.

## Material and Methods

**Study design and ethics**: This study was a single-blind parallel randomized clinical trial (the research participants blinded to randomization). Because of the nature of the study, investigators were not blinded; however, outcome assessors and data analysts were blinded. The study has been registered with the Pan African Clinical Trial Registry (PACTR201906776769795). Any change in the study protocol was updated in the clinical trial registry. By the Declaration of Helsinki, ethical approval was sought and obtained from the Health Research and Ethics Committee of Lagos University Teaching Hospital (LUTH), Idiaraba, Lagos State, Nigeria (Registration Number: ADM/DCST/HREC/2132).

**Sample size and study population**: The sample size was calculated using the G*Power version 3.9.1. The Effect Size (ES) used for calculating the sample size was obtained from the previous study (19) using the International Index of Erectile Function (IIEF-5) primary outcome. The probability level (α), the power (p) and the Effect Size (ES) used for the calculation were then set at 0.05, 0.95 and 3.3 respectively which yielded a sample size of 4 participants per group (total sample size was 8) using independent ttest for between-group analysis. The sample size was increased to 30 to obtain better treatment effects.

The targeted population included patients diagnosed with ED and attending urology and Physiotherapy Outpatient Clinics of LUTH. All the patients were said to have been diagnosed more than six months before the commencement of the study as stipulated in the 5-Items version of the International Index of Erectile Function (IIEF-5).

**Eligibility criteria**: Fourty five (45) male participants, between the ages of 25 to 65 were recruited. The inclusion criteria included participants who have stable medical conditions and diagnosed with ED due to; 1) Neurogenic causes—10(22.2%), 2) Venous occlusion or arterial insufficiency—11 (24.4%), 3)Psychogenic causes—8(17.8%), and 3) Electric shock—1(2.2%).

The exclusion criteria included participants without stable medical conditions and diagnosed with ED due to; 1) Hormonal causes―5(11.1%), 2) Diabetes and renal diseases―10(22.2%). Other exclusion criteria included; participants with priapism, cardiac pacemaker, history of psychiatry or psychological disorders, penile skin lesion/ulcers, or uncontrolled high blood pressure. Additionally, patients on other oral medications were given 2 weeks washout period. In the end, 30 patients were included in the study.

**Role of funding source**: This study did not receive any funding and no organization played any role in the design, conduct, or reporting of this study.

**Data collection instruments**: The instruments used during this study were treadmill machine (an exercise machine for running or walking while staying in one place), Electrical stimulator machine, and IIEF-5. The electrical stimulator is a device that uses electric current to stimulate the nerves for therapeutic purposes. The current is normally produced when the machine is connected to the source of electricity. It has different modes of treatment which include faradic and galvanic currents, and two electrodes (active and inactive). The electrodes are normally applied to the skin surface of the affected area.

The IIEF-5 is an instrument used to determine the presence and extent of ED (23). This Questionnaire consists of only five questions and each IIEF-5 item is scored on a five-point ordinal scale. A response of 1 indicates the least sexual function, whereas a response of 5 indicates the highest sexual function. The highest possible cumulative score for the IIEF-5 is 25, while the least score is 1. A score above 21 was considered as a normal erectile function and a score at or below this value was considered as ED. Overall, according to this scale, ED was classified into four categories: severe (1–7), moderate (8–11), moderate to mild (12–16), mild (17–21), and no ED (22–25) (23).

**Outcomes assessment**: The IIEF-5 questionnaires were given to the participants (self-administered) to assess the level of their ED before the commencement of the treatment. This was repeated at 6 weeks post-intervention. For those subjects that could not read the questionnaire, research assistants interpreted it to them.

**Figure 1:**
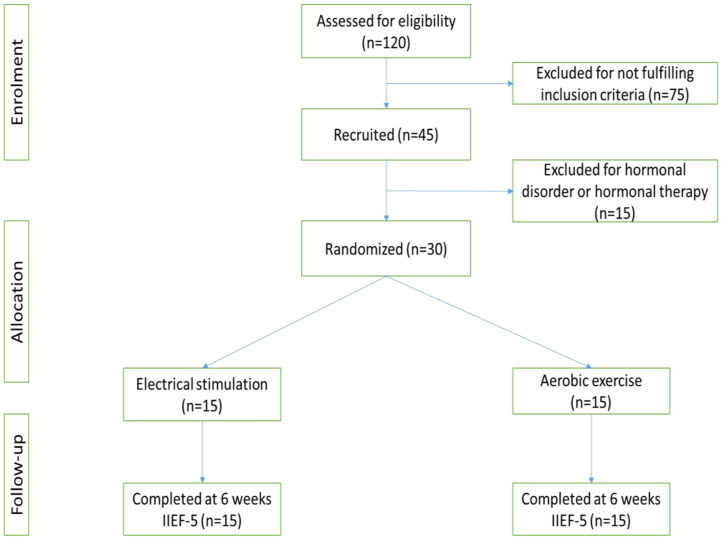
Experimental flow chart

**Randomization and concealment**: Eligible participants who provide informed consent were randomized into one of two treatment groups; Electrical Stimulation (ES) or Aerobic Exercise (AE). A randomization timeline was prepared by a research assistant who did not have communication with any participant throughout the trial and was unaware of the recruitment, screening, assessment, enrolment or treatment process. The randomization series was created by the use of SAS 9.4 statistical software (Cary, NC, USA) with the participants likely to be assigned to a group with an equal chance of allocation. See Figure 1 for the study flow chart.

**Intervention procedures**: The participants were randomly assigned to two experimental groups namely; group A and B, using sealed envelopes, with each group having fifteen (15) participants. Participants in Group A received electrical stimulation, while those in group B received aerobic exercise. The full intervention procedures were described below:

**Group A (Electrical Stimulation)**: The participants were positioned in supine lying and the areas to be treated were adequately exposed. The treatment was carried out by the use of an electronic muscle stimulator (α-wave healthtronic, BM-1006, serial no: C2018-6016, Cybermed Inc. 1618 Valdorast., Davis, CA 95618, USA), using the Erect-fit Stimulation System with two monopolar self-adhesive electrodes. The active electrode was circularly placed on the penis and the inactive electrode at the central-sacral region of the spine (the origin of pelvic plexus). The proper arrangement was made so that the current could be transferred to the inner muscular system, and the intensity was increased to the maximum tolerance level of the participants. The stimulation was carried out for 30 minutes using the following parameters: frequency of 5 Hz, a pulse width of 150µs, and contraction time of 3 seconds (11). Two treatment sessions were given per week for 6 weeks.

**Group B (Aerobic Exercise)-Treadmill**: Roger black treadmill machine, with serial number A0013863; and power of AC220V+10% 50/60 Hz and input of 900V was used for this study. It has a menu which consists of buttons for the following parameters: distance, pulse, speed, time, calories, and start menu. The exercise protocol was based on the guidelines by the American College of Sports Medicine in terms of improving peak Vo2 (55% – 90% of maximal heart rate V02 or 40%–85% of heart rate reserve; 3 – 5 days, 20 – 30 minutes per session) (24). Two treatment sessions were given for 30 minutes each per week for 6 weeks.

**Data analysis**: Descriptive statistics of means and standard deviations were used to summarize the demographic and clinical parameters of the participants. Shapiro-Wilk test was used to determine the normality of the data. Following normal distributions, the data were statistically analyzed using dependent and independent t-tests to find out the difference between the pre- and post-intervention scores of the IIEF-5 within and between the two groups respectively. Independent t-test was also used to compare participants at baseline. All the statistical analysis was performed using Statistical Package for Social Science- SPSS (Windows Version 20.0) at an alpha level of 0.05.

**Data sharing statement**: All participants data collected during the trial will be shared after de-identification. The study protocol, statistical analysis plan, and informed consent form will also be shared. All this information will be shared via a link to be provided immediately after publication so that the data will be available to anyone who wishes to assess it. All data and publication records will equally be updated in the Pan African Clinical Trial registry.

## Results

Thirty (30) patients with ED participated in this study with the age range of 25 to 65 years (mean of 39.17 ± 6.21 years). Details of the demographic and clinical parameters of the study participants are presented in Table 1.

**Table 1:** Demographic and Clinical Parameters of the Participants at Baseline

Groups	Age	Weight (kg)	Height(m)	BMI (kg/m2)	IIEF-5 Scores
	Mean (SD)	Mean (SD)	Mean (SD)	Mean (SD)	Mean (SD)
Group A (N=15)	39.83 (7.65)	75.67 (18.71)	1.65 (0.06)	27.62 (5.90)	11.17 (1.72)
Group B (N=15)	38.50 (5.00)	84.00 (17.32)	1.62 (0.83)	32.24 (8.98)	10.67 (1.63)
*P-Value*	0.067	0.597	0.871	0.075	0.063

N = Number of participants; SD = Standard Deviation; Group A= Electrical stimulation; Group B= Aerobic exercise; BMI= body mass index; IIEF-5=International Index of Erectile Function

The within-group differences are presented in Table 2. The result indicated that the comparison of mean IIEF-5 scores before (11.17±1.72) and after (20.83±1.83) electrical stimulation (Group A) revealed a significant difference (p<0.05). Similarly, there was a significant difference (p<0.05) between patients' IIEF-5 means scores before (10.67±1.63) and after (14.33±2.07) aerobic exercise (Group B). In both the two groups, the post-intervention IIEF-5 scores had the highest means.

**Table 2:** Comparison of Pre- and Post-intervention IIEF-5 Scores for within Group Differences

Variables		Pre-Intervention	Post-Intervention	t-value	p-value
		Mean (SD)	Mean (SD)		
IIEF-5 score	Group A	11.17 (1.72)	20.83 (1.83)	-29.00	0.001**
	Group B	10.67 (1.63)	14.33 (2.07)	-6.33	0.001**

** Significance at p-value<0.05

Group A= Electrical stimulation; Group B= Aerobic exercise; IIEF-5=International Index of Erectile Function

The between-group difference was presented in Table 3. The result indicated that there was a significant difference (p<0.05) when post-intervention mean IIEF-5 score of group A (20.83±1.83) was compared with that of group B (14.33±2.07). This also indicated that Group A was better than Group B in terms of treatment effect.

## Discussion

The findings of this study indicated that both electrical stimulation and aerobic exercise are effective non-invasive treatment options for ED. On direct comparison, however, electrical stimulation proved to be of superior effectiveness. All the important characteristics of the participants such as BMI, age, the duration of ED and IIEF-5 scores, which could have affected the findings of this study, were similar at baseline. This validated the outcomes of the study as being solely due to the effect of the interventions.

Concerning the effect of aerobic exercise on ED, our finding has corroborated with those of the previous studies (12–16). The physiological basis of the therapeutic effect of aerobic exercise on ED in this study could be attributed to both acute and long-term changes in the blood vessel walls (12). The immediate vascular relaxation following physical activity is due to body warming effects; decrease in nerve activities; local production of certain chemicals such as lactic acid, and nitric oxide (NO), and changes in certain hormones and their receptors (25). The repetitive physical activity-induced increased blood flow and vascular shear stress lead to substantial remodeling of the vascular system (26). This adaptive response alters the endothelium by increasing the expression of nitric oxide synthase mRNA; resulting in increased synthesis of NO and improved endothelial function (27). Although most of the adaptive changes are limited to the working muscles, the endothelial functional improvement seems to be the entire body response to exercise (26). For example, Belardinelli et al. (28) reported a significant improvement in flow-mediated dilation of brachial artery in the group of patients with ED subjected to 8-weeks aerobic exercise compared to controls.

Another possible physiological basis of our finding was the improvement in the testosterone concentration in the body. Even though testosterone level has been reported to rise after resistant training (29), a recent randomized controlled trial involving animal models revealed that the normal plasma level of testosterone was completely restored in a group of high-fat diet (HFD) fed rabbits with metabolic syndrome-induced hypogonadotropic hypogonadism and erectile dysfunction, following a 12-week treadmill training compared to controls (30). Analysis of penile gene expression indicates that physical activity increases genes related to a smooth muscle phenotype along with NO formation and signaling (30). Since erectile tissue function is finely regulated by a healthy smooth muscle tissue whose relaxant or contractile mechanisms are dependent on NO signaling and formation (31), the improvement in ED seen in our participants may not be unrelated to this these mechanisms. In addition, considering that NO signaling within the penis is also androgen-dependent (32), it is possible to conceive that the aforementioned findings in our participants are related to the restoration of normal androgen levels induced by physical exercise. Thus, we believe that the improvement in erectile function seen in our patients following treadmill exercise may be attributed to the increase in both NO synthesis and testosterone levels.

On the other hand, the present study reported significant improvement in erectile function following electrical stimulation. This is consistent with the findings of previous studies. For instance, Carboni et al. (19) reported improvement in erectile function in individuals with ED following 4-weeks of functional electrical stimulations. A similar finding has been reported by Kayigil et al. (33) and Van Kampen et al. (18), who concluded that electrical stimulation plays an important role in the rehabilitation of ED. It will be pertinent to note that electrical stimulation is also effective in the management of neurogenic ED. For example, Fayiz et al. (11) conducted a study to determine the effectiveness of transcutaneous electrical stimulation in the rehabilitation of patients with post-prostatectomy ED. They reported significant improvement in erectile function of the patients compared to controls. A similar finding has been reported by Derouet et al. (34). These were supportive of our findings considering the fact that many studies excluded this group of patients from their research envisaging that electrical stimulation will not affect neurogenic ED. Nevertheless, the possible physiologic rationale behind the positive effect of electrical stimulation in the current study could be the already established ability of this modality to induce regeneration of cavernous sinusoidal endothelium smooth muscle, with increased NO release (35,36). It was further hypothesized that the increased penile strength and the resulting gain in muscular mass will improve the venous occlusion mechanism leading to required filling of corpus cavernosal bodies with blood (20,21). Concerning the positive effect of electrical stimulation on neurogenic ED, it may be possible that the electric currents have spread to the deeply situated cavernosal nerves. This is possible since a very low stimulating frequency of 5Hz was used in the study and the inactive electrode was placed on the sacral region of the spine, the origin of pelvic plexus. This may constitute part of the reasons for the effectiveness of our intervention. An animal study has demonstrated the ability of electrical stimulation to rescue erectile function after cavernosal nerve injury by inducing nerve regeneration through pericyte (PC)-derived exosome (37). Pericytes are bionanoparticles that are distributed in the erectile tissue and play important roles in the regulation of penile erection, including promoting angiogenesis and neurogenesis through interacting with endothelial cells. Even though all the participants in the current study have intact cavernous nerve, the electric current from our intervention might have led to an increase in intracavernous pressure (38).

The most important finding of the current study is the superiority of electrical stimulation over aerobic exercise in the management of ED, which did not conform to our initial hypothesis. This finding could be attributed to the multifactorial nature of the etiologies of ED. Based on its causes, ED has been classified into vasculogenic, neurogenic, hormonal, or psychogenic. Vasculogenic ED is the one that presents with vascular endothelial dysfunction, though ED is generally considered to be an early indicator of cerebrovascular disease. Aerobic exercise seems to be more effective in the rehabilitation of vasculogenic ED due to its ability to restore endothelial function. Moreover, the long-term adaptive changes such as an increase in the expression of nitric oxide synthase occur only with chronic exercise. In the present study, almost half (46.6%) of the participants were diagnosed with vasculogenic ED and might have derived more benefit from exercise intervention than the remaining participants. Contrary to this, electrical stimulation can provide the local and more immediate effect to all forms of ED through its effect on cavernosal smooth muscle and nerve.

In conclusion, this study indicated that electrical stimulation has been proven to be more effective than aerobic exercise in the treatment of individuals with erectile dysfunction. This study was conducted on a few participants (30) diagnosed with ED due to the lack of funding for the research. Furthermore, long-term follow-up was not conducted because of time constraint. Future studies may, therefore, include large sample sizes and conduct sufficient follow-ups to see the long-term effects of the treatments.
